# Unlocking the potential of *Rosa roxburghii* Tratt polyphenol: a novel approach to treating acute lung injury from a perspective of the lung-gut axis

**DOI:** 10.3389/fmicb.2024.1351295

**Published:** 2024-01-11

**Authors:** Li Tang, Shuo Zhang, Min Zhang, Pengjiao Wang, Guiyou Liang, Zhitong Gan, Xiuli Gao

**Affiliations:** ^1^State Key Laboratory of Functions and Applications of Medicinal Plants, School of Pharmaceutical Sciences, Guizhou Medical University, Guiyang, China; ^2^Microbiology and Biochemical Pharmaceutical Engineering Research Center of Guizhou Provincial Department of Education, Guizhou Medical University, Guiyang, China; ^3^School of Chinese Ethnic Medicine, Guizhou Minzu University, Guiyang, China; ^4^Translational Medicine Research Center, Guizhou Medical University, Guiyang, China

**Keywords:** *Rosa roxburghii* Tratt polyphenol, acute lung injury, gut homeostasis, short-chain fatty acids, *Akkermansia muciniphila*

## Abstract

**Introduction:**

Acute lung injury (ALI) is a serious respiratory disease characterized by progressive respiratory failure with high morbidity and mortality. It is becoming increasingly important to develop functional foods from polyphenol-rich medicinal and dietary plants in order to prevent or alleviate ALI by regulating intestinal microflora. Rosa roxburghii Tratt polyphenol (RRTP) has significant preventive and therapeutic effects on lipopolysaccharide-induced ALI mice, but its regulatory effects on gut homeostasis in ALI mice remains unclear.

**Methods:**

This study aims to systematically evaluate the ameliorative effects of RRTP from the perspective of “lung-gut axis” on ALI mice by intestine histopathological assessment, oxidative stress indicators detection and short-chain fatty acids (SCFAs) production, and then explore the modulatory mechanisms of RRTP on intestinal homeostasis by metabolomics and gut microbiomics of cecal contents.

**Results:**

The results showed that RRTP can synergistically exert anti-ALI efficacy by significantly ameliorating intestinal tissue damage, inhibiting oxidative stress, increasing SCFAs in cecal contents, regulating the composition and structure of intestinal flora, increasing Akkermansia muciniphila and modulating disordered intestinal endogenous metabolites.

**Discussion:**

This study demonstrated that RRTP has significant advantages in adjuvant therapy of ALI, and systematically clarified its comprehensive improvement mechanism from a new perspective of “lung-gut axis”, which provides a breakthrough for the food and healthcare industries to develop products from botanical functional herbs and foods to prevent or alleviate ALI by regulating intestinal flora.

## 1 Introduction

Acute lung injury (ALI) is a serious respiratory disease characterized by progressive respiratory failure with high morbidity and mortality. Currently, in addition to respiratory support, ALI lacks effective medication (Gorman et al., 2022). Oxidative stress, a crucial factor in the pathological progress of ALI, is pivotal for maintenance of tissue homeostasis and protection against infections (Ornatowski et al., 2020). A high level of reactive oxygen species (ROS) could damage microvascular barriers and aggravate pulmonary edema by exceeding the body’s scavenging capacity (Meng et al., 2022).

Intestinal microbiota and their metabolites affect antibacterial action, immunoregulation and nutrient metabolism of human body (Huang et al., 2019; Iddrisu et al., 2022). Gut microecological imbalance, related to pulmonary disease, can disrupt the integrity of the intestinal barrier, activate the systemic immune system, and aggravate immune damage to the lung. This interaction between the lung and gut is called the “lung-gut axis” (Wedgwood et al., 2020). Therefore, maintaining intestinal homeostasis is essential to ameliorating lung disease. Short-chain fatty acids (SCFAs) are generated by symbiotic intestinal flora. As a vital energy source for intestinal epithelial cells, they play a significant role in maintaining microecological homeostasis of intestine and immune balance of host, and modulating gut pH value, and are “star” molecules which regarded as potential novel targets for ALI therapy (de Vos et al., 2022; Hou et al., 2022; Huang et al., 2022).

Functional foods, including flavonoids, polyphenols, terpenoids and other natural active ingredients, have the properties of regulating oxidative stress, protecting lung and ameliorating the disturbance of intestinal flora (He et al., 2021). Consequently, the food and healthcare industries are increasingly focusing on developing products to prevent or alleviate ALI by regulating intestinal flora from the plant-based functional herbal medicine and foods.

*Rosa roxburghii* Tratt (RRT) is an underutilized nutritional crop from the Rosaceae family with high medicinal properties and primarily distributed in the mountainous areas in southwest China. Phytochemical analyses confirmed that RRT contains organic acids, flavonoids, polyphenols and other natural active ingredients, with functional activities incomparable to other fruits (Wang et al., 2021). RRT exhibits a diversity of biological characteristics against immune disorder, inflammatory disease and tumors, and can also regulate gut microbiota dysfunction caused by hyperlipidemia and diabetes (Wang et al., 2020; Ji et al., 2022). RRT polyphenol (RRTP), including phenolic acids, coumarins, lignans, tannins, and flavonoids, is one of the most important active components of RRT. In our previous study, we confirmed that RRTP has a significant preventive and therapeutic effect on ALI mice induced by lipopolysaccharide (LPS) (Tang et al., 2022, 2023), and it is a functional food resource with great potential for adjuvant therapy of ALI. In addition, the intestinal flora of ALI mice has been found to be dysfunctional (Tang et al., 2021), and plant-based foods containing polyphenol have also been shown to alleviate inflammatory diseases by regulating intestinal homeostasis (Wu et al., 2021).

Accordingly, in order to further reveal the amelioration mechanism of RRTP on ALI, we hypothesized that RRTP had an intervention effect on intestinal microflora of LPS-treated ALI mice in this study. This intervention effect may shed light on adjuvant and complementary therapy development for ALI. To validate this hypothesis, we systematically estimated the effects of RRTP on intestinal tissues of ALI mice through intestine histopathological assessment, oxidative stress indicators detection and SCFAs production, and then explored the modulatory mechanisms of RRTP on intestinal homeostasis by metabolomics and *gut microbiome* of cecal contents.

## 2 Materials and methods

### 2.1 Reagents and chemicals

According to our previous research (Tang et al., 2022), RRTP is extracted from RRT fruit and preserved in microbiology and biochemical pharmaceutical engineering research center of Guizhou Medical University. Lipopolysaccharide (LPS, *Escherichia coli serotype* 055:B5) was purchased from Sigma-Aldrich. Acetonitrile and methanol (UPLC-grade) were collected from Merck.

### 2.2 Animals and drug administration

**Figure 1:**
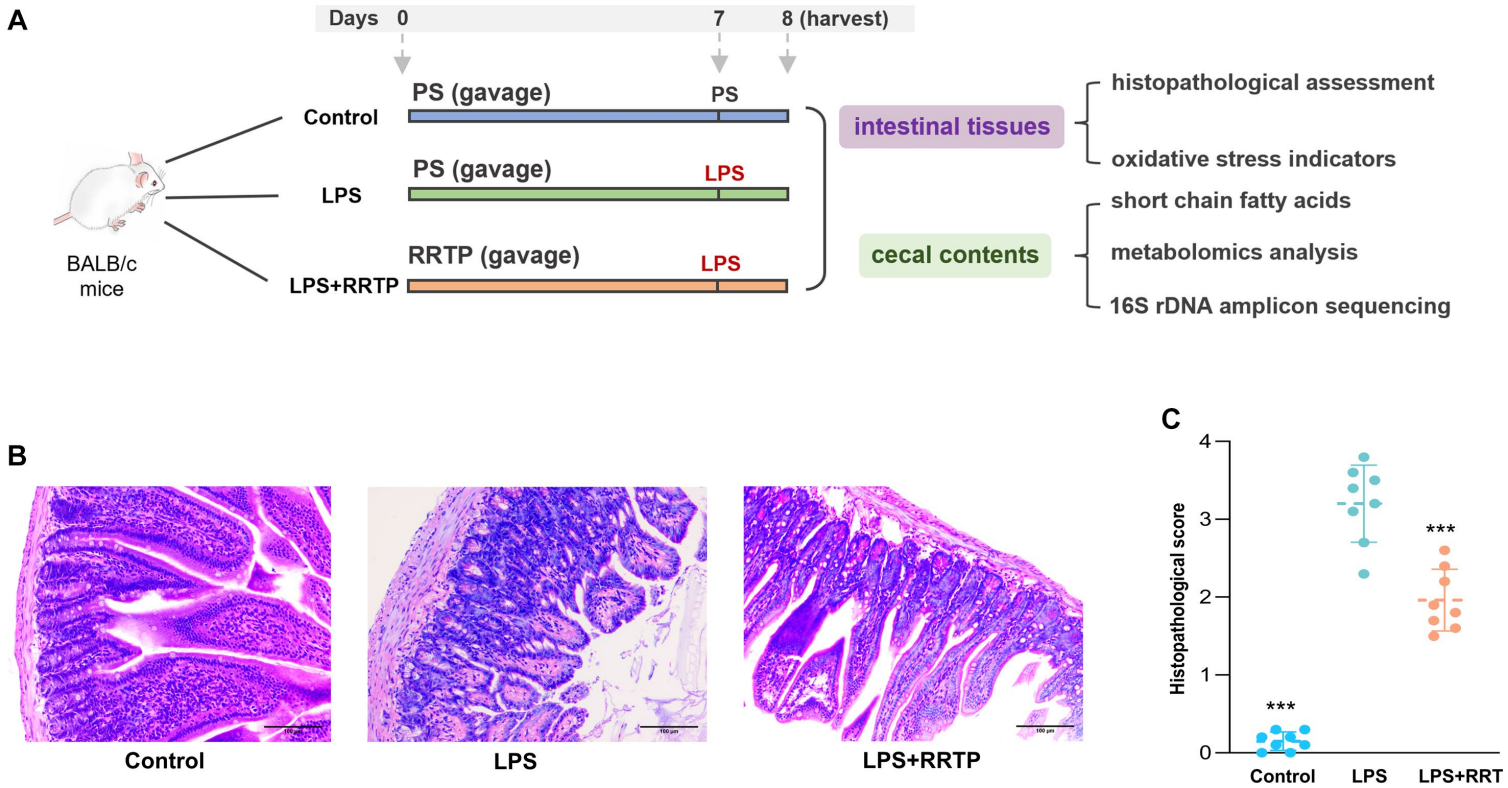
Comprehensive evaluation of RRTP on intestinal homeostasis in ALI mice. **(A)** Schematic of animal experiment protocols. PS: physiological saline. LPS: lipopolysaccharide. **(B)** Representative micrographs of histological sections of the gut tissues in LPS-induced ALI mice. (H&E staining, original magnification ×200). **(C)** Pathological score of histological sections. *Compared with the model group, ***P < 0.001.

All animal experiments were performed in accordance with the Animal Care and Use Guidelines of Guizhou Medical University, and the protocols were reviewed and approved by the Animal Ethics Committee of institution (License No. SYXK (Qian) 2018-0001). Specific pathogen-free (SPF) male BALB/c mice were obtained from Changsha Tianqin Biotechnology Co., Ltd. (License No. SCXK (Xiang) 2019-0013, Hunan, China), and acclimatized in a standard mice laboratory with free access to water and food for 7 days. Animals were performed daily administration of physiological saline or RRTP. RRTP extracts dissolved in physiological saline gavage for 7 days at 10 mL/kg/d. Thirty minutes after the last administration, mice were anesthetized by intraperitoneal injection (10% chloral hydrate), and LPS (2 mg/kg) was administered by oropharyngeal instillation to establish ALI mice. Instead of LPS, physiological saline was given to the normal group. After the addition of LPS, the mice were sacrificed 24 h later, and gut tissues and cecal contents were collected and stored at −80°C for further analysis. The schematic of animal experiment protocols was depicted in Figure 1A.

### 2.3 Histopathological assessment

The gut tissues were washed with PBS, fixed with 4% paraformaldehyde, dehydrated with ethanol, embedded with paraffin wax and cut into 4 μm thick sections. After successive staining with hematoxylin-eosin (H&E), histopathological alteration was evaluated with an optical microscope.

### 2.4 Determination of oxidative stress indicators

A supernatant was obtained for biochemical measurement of intestinal tissues after homogenization and centrifugation in cold PBS, and its protein concentration was detected according to the manufacturer’s instructions. The activity of glutathione (GSH), myeloperoxidase (MPO), superoxide dismutase (SOD) and malondialdehyde (MDA) in gut tissues were measured to evaluate the degree of oxidative stress damage in ALI mice. Oxidative stress detection kits were purchased from Nanjing Jiancheng Bioengineering Institute (Nanjing, China).

### 2.5 Measurement of short chain fatty acids

SCFAs were extracted from cecal contents with 1 mL aqueous solution. After centrifugation at 10,000 rpm for 10 min, the supernatant was collected, and SCFAs were extracted from the supernatant with 1 mL diethyl ether. Finally, the diethyl ether solution was detected by gas chromatography (GC).

The separation of SCFAs was carried out with GC system (7890B, Agilent) equipped with a DB-WAX capillary column (30 × 0.53 mm, 1 μm, Agilent). The temperature of injection port and detector were 270°C and 280°C, respectively. Injection volume was 2 μL. The initial temperature of the capillary column was set at 100°C for 1 min and then increased to 150°C at a rate of 5°C per minute for 7 min. The quantification of acetic acid, propionic acid and butyric acid was conducted by comparison with chemical standards.

### 2.6 Cecal contents metabolomics analysis

Endogenous metabolites in cecal contents were detected by UHPLC-Q-Exactive Plus Orbitrap-MS (Thermo Fisher Scientific). Briefly, 50 mg cecal contents was extracted with the precooled mixture of acetonitrile, methanol and aqueous solution (2:2:1). After homogenization, vortex and centrifugation at 4°C for 20 min, the supernatant was filtered through 0.22 μm membrane for metabolomics analysis.

The separation of metabolites was conducted on a 1.8 μm C_18_ column (2.1 × 100 mm, ZORBAX Eclipse Plus, Agilent, United States) with flow rate was 0.3 mL/min. The optimized gradient elution procedure (phase A: 0.1% formic acid H_2_O; phase B: 0.1% formic acid acetonitrile) was as follows: 0–2.5 min, 2–2% B; 2.5–5 min, 2–40% B; 5–12 min, 40–100% B; 12–16 min, 100–100% B; 16–16.1 min, 100–2% B; 16.1–19 min, 2–2% B. Temperature of capillary and aux gas heater were 320°C and 350°C, respectively. Ion spray voltage was 3.5/2.8 kV (+/−), S-lens RF level was 50, dynamic exclusion was 3 s, and the data was analyzed in both negative and positive modes from 100 to 1,500 *m*/*z*. Compound Discoverer 3.2 was used to pretreat MS data, including retention time correction, peak matching and peak recognition. Primary and secondary mass spectrum fragment information from KEGG and HMDB databases was used to identify metabolites. In addition, components were validated by MS^2^ spectroscopy using an internal metabolite spectrum library. An analysis of intestine-related metabolic pathways implicated in ALI after RRTP preventive treatment was carried out using MetabolAnalyst 5.0. Metabolic pathways with an impact value greater than 0.1 were considered significant.

### 2.7 16S rDNA amplicon sequencing

A CTAB method was used to extract genome DNA from cecal contents samples and 1% agarose gels were used to verify DNA purity and concentration. Bacterial 16S rDNA gene was amplified by PCR using specific primers. All PCR reactions were carried out with DNA template (10 ng), forward and reverse primers (2 μM) and Phusion^®^ High -Fidelity PCR Master Mix (15 μL, New England Biolabs). During thermal cycling, initial denaturation was carried out at 98°C for 1 min, followed by 30 cycles of denaturation for 10 s, annealing for 30 s, and elongation for 30 s. PCR products were purified with Gel Extraction Kit (Qiagen, Germany). The sequencing libraries were prepared following the manufacturer’s instructions using TruSeq^®^ DNA PCR-Free Sample Preparation Kit (Illumina, United States) and index codes were also added to the libraries. Finally, Illlumina NovaSeq platform was used to sequence the library and generate 250 bp paired-end reads.

### 2.8 Statistical analysis

A statistical analysis was carried out using GraphPad Prism 9.0 and SPSS 23.0. For metabolomics analysis, ANOVA and student’s *t*-test were used to evaluate differences, with *p* < 0.05 considered significant. R packages (2.15.3) and QIIME were used for 16S rDNA amplicon sequencing analysis. Alpha diversity, including ACE (Abundance-based Coverage Estimator), observed-species, Chao1, and PD_whole_tree, were evaluated using OTU table in QIIME. PCA analysis in beta diversity was performed using the ade4 package and ggplot2 package in R to reduce the dimension of the original variables.

## 3 Results and discussion

### 3.1 RRTP alleviated pathological injury of intestinal tissues

Intestinal tissues were evaluated using H&E for morphological and histological changes. After physiological saline inhalation, the intestinal tissues of normal mice showed no significant histological changes (Figure 1B). Interestingly, in LPS-treated ALI mice, the gut tissues showed significant inflammatory zinfiltration, congestion as well as gut villus damage in the model group. It should be noted that after early intervention with RRTP, histopathological score of gut tissues in ALI mice was significantly decreased (Figure 1C), and the severity of pathological changes was significantly attenuated. Therefore, RRTP-mediated intestine-protection may be essential for maintaining intestinal tissues morphology.

### 3.2 RRTP attenuated oxidative stress damage of intestinal tissues

**Figure 2:**
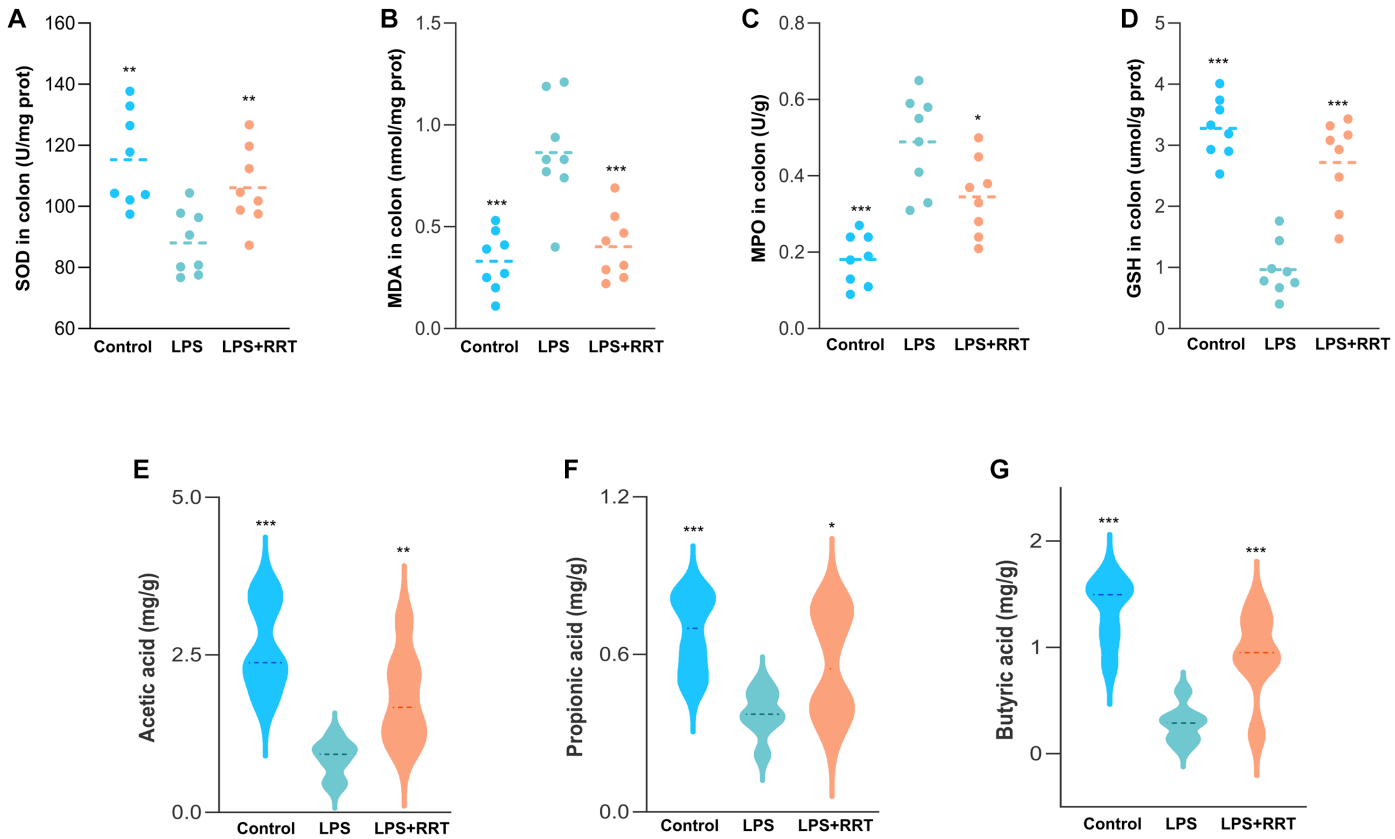
Comprehensive evaluation of RRTP on oxidative stress and SCFAs in ALI mice. **(A-D)** The levels of SOD, MDA, MPO and GSH. **(E-G)** Analysis of SCFAs in the cecal contents of the ALI mice. *Compared with the model group, *P < 0.05, **P < 0.01, ***P < 0.001.

Excess ROS can cause the peroxidation of unsaturated fatty acid in the lipid layer of cell membranes, which stimulates neighboring epithelial or endothelial cells and amplifies tissue damage (Wang et al., 2020). Therefore, uncontrolled and prolonged oxidative stress response increases the risk of tissue injury in multiple organs, a characteristic feature of several inflammation-related lung diseases (Kawanishi et al., 2017). Accordingly, quantitative measurement of representative oxidation-related indicators of SOD, MPO, MDA and GSH in intestine samples is helpful to reveal whether RRTP can maintain intestinal homeostasis by alleviating oxidative stress response in intestinal tissues to achieve the prevention and treatment of ALI. The levels of oxidative stress indicators in the model group and the normal group were significantly different, indicating that the oxidative stress response was disturbed in the intestinal tissues of ALI mice. Notably, the increase of MPO and MDA and the decrease of SOD and GSH were remarkably restrained in ALI mice after early intervention with RRTP (Figures 2A–D), suggesting that RRTP played a direct role in the ALI therapy by inhibiting over-activated oxidative damage to maintain intestinal homeostasis.

### 3.3 RRTP strengthened the intestinal barrier by regulating SCFAs

SCFAs, one of the most important metabolites produced by intestinal flora, are a class of organic fatty acids with no more than 6 carbon atoms, mainly including acetic acid, propionic acid and butyric acid. Many studies have confirmed that SCFAs, particularly butyric acid, can maintain intestinal homeostasis and the dynamic balance of oxidation-antioxidant response, enhance intestinal barrier (Wang et al., 2022), and they can also reduce inflammation by activating immune cells and epithelial cell receptors after entering lung tissue through blood circulation (Dang et al., 2019; Yoo et al., 2020). Consequently, we detected the SCFAs concentration in the cecum of ALI mice to investigate whether the mitigatory effects of RRTP on ALI was connected with the production of SCFAs. Our results manifested that the SCFAs concentration in cecal contents of LPS-treated ALI mice was lower than that of the normal mice, and after early intervention with RRTP, the cecal SCFAs contents of ALI mice was significantly increased (Figures 2E–G). These results revealed that RRTP strengthened the intestinal barrier by increasing the contents of intestinal SCFAs to achieve a palliative effect of RRTP on ALI.

### 3.4 RRTP improved cecal contents metabolic disorders

**Figure 3:**
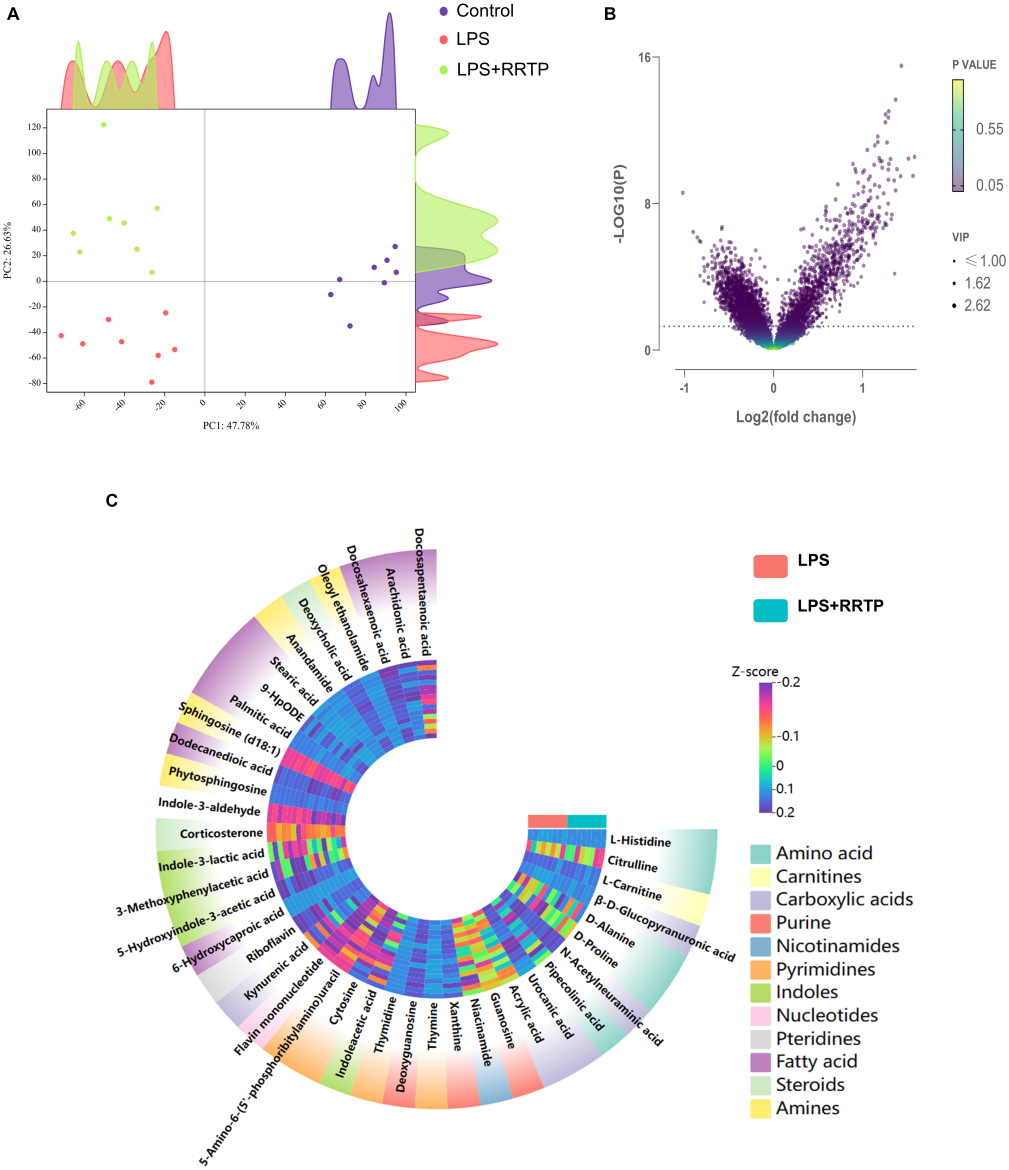
Metabolomics analysis of RRTP on the cecal contents in ALI mice. **(A)** PCA of metabolomics data in the three groups. **(B)** Volcano plot of endogenous metabolites in the model group and the RRTP group. **(C)** Heatmap of 40 differentially expressed metabolites (DEMs).

**Figure 4:**
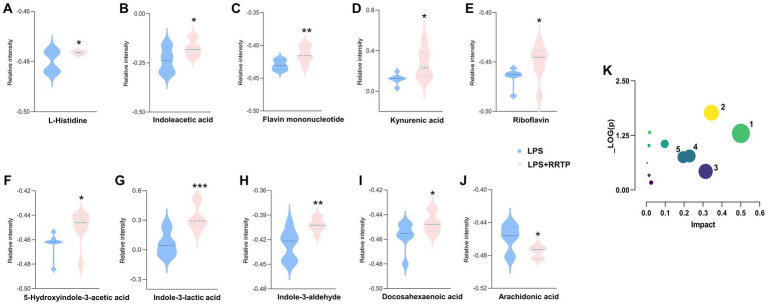
Metabolomics analysis of RRTP on the cecal contents in ALI mice. **(A-J)** The expression level of ten representative metabolites with significant difference between the model group and the RRTP group. **(K)** Metabolic pathways analyzed by MetaboAnalyst. 1: Riboflavin metabolism; 2: Histidine metabolism; 3: Arachidonic acid metabolism; 4: Arginine biosynthesis; 5: Nicotinate and nicotinamide metabolism. *P < 0.05, **P < 0.01, ***P < 0.001.

Metabolic profiling data were analyzed without grouping using PCA to determine cecal contents samples distribution and the model’s reliability. Compared with the model group, RRTP metabolomics data showed good separation without molecule selection, suggesting that RRTP have significant intervention effects on cecal contents metabolic disorders in ALI mice (Figure 3A). Supervisory pattern OPLS-DA was applied to identify differences in metabolic profiling between the two groups (Supplementary Figure S1), and its degree of reliability and fit was determined by 200 permutations. The results showed that *Q*^2^ and *R*^2^ were less than the original value, and the intersection of the vertical axis with *Q*^2^ regression line was also less than 0 (Supplementary Figure S2). In general, the pattern was reliable, stable, and not overfit, making it suitable for metabolites prediction. Differentially expressed metabolites (DEMs) were defined as variables that significantly contributed to grouping (*p* < 0.05, VIP >1) (Figure 3B). According to the metabolomics of cecal contents, RRTP reversed 40 endogenous metabolites, of which 30 were upregulated and 10 were downregulated (Figure 3C). Among these biomarkers, more than 50% were indoles and their derivatives, amino acids and lipids (Figures 4A–J), and the endogenous metabolites were markedly enriched in five metabolic pathways, including riboflavin metabolism, histidine metabolism, arachidonic acid metabolism, arginine biosynthesis and nicotinate and nicotinamide metabolism (Figure 4K). The results indicated that RRTP modulated the abnormal amino acid metabolism and lipid metabolism of intestine in ALI mice.

### 3.5 RRTP alleviated the disturbance of gut microbiota

In recent years, the imbalance of intestinal flora has highlighted its role as a pivotal factor of a variety of acute and chronic pulmonary inflammatory diseases, and the disorder of gut microbiota can induce or promote the development of pulmonary inflammation (Frati et al., 2018). The holistic structural alteration of intestinal flora in response to RRTP were illustrated by analysis of the 16S rDNA gene sequencing of microorganic samples isolated from the cecal contents of the normal, model and RRTP mice.

**Figure 5:**
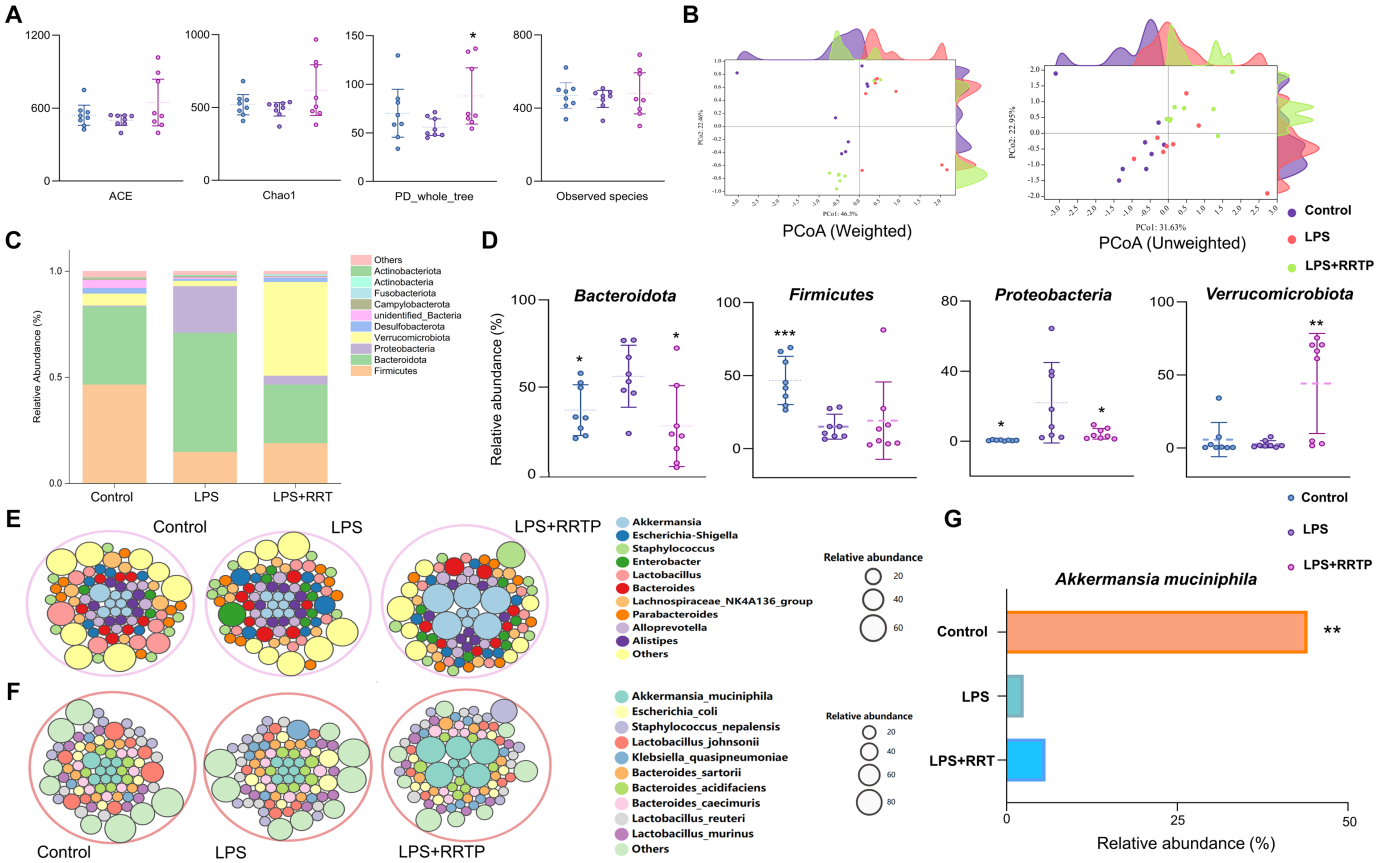
Systematic assessment of RRTP on gut microbiome diversity and structure in ALI mice. **(A)** Alpha diversity differences were estimated by the ACE, Chao1, PD_whole_tree, and observed species indices. **(B)** PCoA plot base of the relative abundance of OTUs showing bacterial structural clustering. Weighted UniFrac PCoA plots. Unweighted UniFrac PCoA plots. **(C)** Component proportion of bacterial phylum in each group. **(D)** Four representative bacteria significantly changed at the phylum level. **(E)** Circle packing diagram of bacteria at the genus level in each group. **(F)** Circle packing diagram of bacteria at the species level in each group. **(G)** The relative abundance of Akkermansia_muciniphila in the three groups. *Compared with the model group, *P < 0.05, **P < 0.01, ***P < 0.001.

To estimate the differences in bacterial diversity between the three groups, sequences were aligned to evaluate alpha diversity and beta diversity. There are differences in ACE, Chao1, PD_whole_tree and observed species among the three groups of samples (Figure 5A). Both weighted and unweighted PCoA analysis of beta diversity demonstrated a degree of separation of the three groups on the basis of first two PCoA (Figure 5B). These results indicated that RRTP consumption improved the species richness and community diversity of intestinal microflora in ALI mice.

Microbial taxon classification was used to determine the relative proportions of the phylum-level of dominant taxa. We noticed considerable variation in intestinal flora of samples in each group. *Firmicutes* (46.68% versus 14.98%), *Bacteroidota* (36.76% versus 56.16%), *Proteobacteria* (0.49% versus 21.96%), and *Verrucomicrobiota* (5.71% versus 2.52%) were enriched in the normal group compared to the model group (Figure 5C). Intriguingly, after early intervention with RRTP, the proportions of *Bacteroidota* and *Proteobacteria* in ALI mice were significantly reduced, while *Firmicutes* and *Verrucomicrobiota* were significantly increased, and this reversal trend was close to the normal group (Figure 5D). Notably, *Verrucomicrobiota* was the most predominant phylum in the RRTP group. This finding attracted the great attention of our team members, and further analysis of microflora composition at genus level and species level is helpful to reveal the intervention effects of RRTP on gut microbiota of ALI mice. The results showed that *Akkermansia* and *Akkermansia muciniphila* (*A. muciniphila*) were dominant in the RRTP group at the genus and species level, respectively (Figures 5E–G).

### 3.6 Correlation analysis

**Figure 6:**
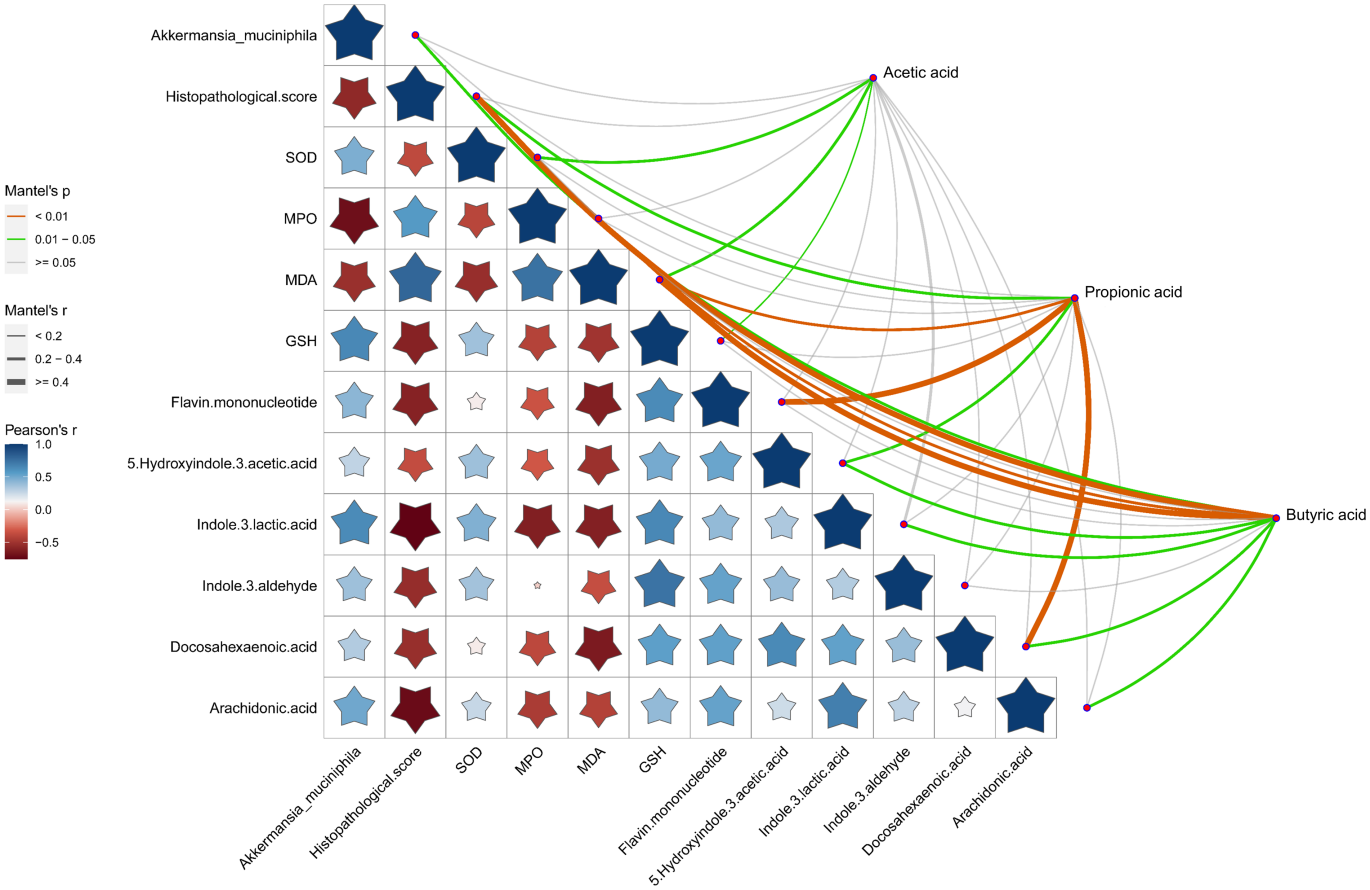
Correlation analysis of SCFAs with Akkermansia muciniphila, pathological score of the gut tissues, oxidative stress indicators and the representative DEMs. The orange color curve indicated the significant correlation, the green color curve indicated the statistical difference, and the gray curve indicated no significant correlation. The thickness of the curve and the size of the pentagram represent the size of the correlation coefficient. The thicker the curve (or the larger the pentagram), the greater the correlation coefficient.

Association analysis of SCFAs with *A. muciniphila*, pathological score of the gut tissues, oxidative stress indicators and the representative DEMs was performed to better understand the comprehensive amelioration mechanism of RRTP on intestinal homeostasis in ALI mice (Figure 6). SCFAs, especially propionic acid and butyric acid, were closely related to the pathological score, oxidative stress indicators and intestinal endogenous metabolites (including indole and its derivatives, fatty acids) of ALI mice, suggesting that SCFAs as an effective messenger may be involved in the role of RRTP in maintaining intestinal homeostasis by alleviating intestinal tissue pathological damage, attenuating oxidative stress response and improving metabolic disorders. Conspicuously, butyric acid production was also associated with *A. muciniphila*, the most prominent dominant bacterium of the RRTP group, and this result accurately elucidated that butyric acid and *A. muciniphila* may play a synergistic role in the complex mechanism of RRTP. In addition, we also noted that *A. muciniphila* was negatively correlated with histopathological score, the concentration of MPO and MDA, and positively correlated with the concentration of SOD, GSH and endogenous metabolites indole derivatives. It is suggested that *A. muciniphila* may inhibit excessive oxidative stress response, reduce intestinal tissue injury and increase intestinal indole derivatives to promote intestinal homeostasis.

### 3.7 Comprehensive regulation of RRTP on intestinal homeostasis in ALI mice

#### 3.7.1 Suppression of oxidative stress response

Lung tissue damage can easily cause hypoxia in local and adjacent tissues, causing the accumulation of reactive oxygen species, leading to oxidative stress, which in turn leads to the occurrence and aggravation of ALI (Kellner et al., 2017). MDA, a lipid peroxidation product, is a common marker of oxidative damage, while MPO is an accurate evaluative indicator of neutrophil infiltration and aggregation in inflammatory diseases, involved in the development of ALI and lipid peroxidation (Wang et al., 2020). GSH can catalyze the reduced glutathione to scavenge ROS, thereby alleviating tissue damage caused by lipid peroxides (such as MDA) (Day, 2009). SOD can relieve tissue damage by removing metabolic byproducts produced by oxidative injury (Miao and St. Clair, 2009). In this study, we found that the activities of MDA and MPO were significantly increased, while SOD and GSH were remarkably decreased in the model group ALI mice. Intriguingly, after early intervention with RRTP, MDA and MPO decreased significantly, while SOD and GSH increased significantly. The RRTP-regulated lipid and amino acid differential metabolites, including docosahexaenoic acid (Tatsumi et al., 2022; Younes et al., 2022), docosapentaenoic acid (Wang et al., 2019) and histidine (Thalacker-Mercer and Gheller, 2020), identified in our metabolomics study, have effects on reducing inflammation and alleviating oxidative stress. Furthermore, *Bacteroidota* is one of the most aerobically tolerant anaerobic bacteria, and its relative abundance increases significantly under ascending levels of oxidative stress in the organism (Zafar and Saier, 2021). It is also involved in the release of toxic products in the process of protein decomposition, aggravating the inflammatory response of the body (Jandhyala et al., 2015). RRTP significantly decreased the relative abundance of *Bacteroidetes* at the phylum level in this study. Accordingly, our results suggested that oxidative stress response of RRTP on intestinal tissues of ALI mice may be achieved through effective modulation of endogenous metabolites and the composition of intestinal flora.

#### 3.7.2 Promotion of *Akkermansia muciniphila* production

*A. muciniphila*, a “sentinel of the gut,” has emerged as a promising candidate probiotic colonizing in the mucous layer. It plays an important role in promoting host metabolic function, repairing intestinal barrier, maintaining intestinal homeostasis to reduce tissue inflammation and oxidative stress (Zhang et al., 2019; Ouyang et al., 2020). Excitingly, we found that *A. muciniphila* was the most significant dominant bacteria in the RRTP group in this study, with a proportion of nearly 50%, indicating that RRTP could maintain intestinal homeostasis by improving the production of *A. muciniphila*. Given that the production of *A. muciniphila* is related to multiple improvements in intestinal functions, it comes as no surprise that early RRTP intervention induced *A. muciniphila* adequacy would provide protection against intestinal tissue injury.

#### 3.7.3 Enhancement of SCFAs concentration

A variety of bacteria under *Firmicutes* belong to probiotics, which are the dominant bacteria producing SCFAs in intestinal flora and can maintain the homeostasis of gut microbiota (Markowiak-Kopeć and Śliżewska, 2020). In addition, it has been confirmed that *A. muciniphila* also produce SCFAs, and that the production of SCFAs, especially butyric acid, increases intestinal barrier integrity, restrains intestinal inflammatory and oxidative stress responses and minimizes bacterial translocation (Wang et al., 2022). RRTP significantly increased the relative abundance of *Firmicutes* at the phylum level in this study. Consequently, our results manifested that RRTP may enhance the production of SCFAs by regulating the composition and structure of gut flora in order to maintain intestinal homeostasis.

#### 3.7.4 Regulation of indole derivatives

Tryptophan metabolites, including indoleacetic acid (IAA), 5-hydroxyindole-3-acetic acid (5-HIAA), indole-3-aldehyde (I3A), indole-3-lactic acid (ILA), and kynurenic acid, can be produced by gut microbiota through tryptophan or kynurenine transformation pathways. These intestinal microbiota-derived tryptophan metabolites play a vital role in maintaining gut homeostasis, as well as attenuating inflammatory-related diseases (Su et al., 2022). I3A can restore mucosal integrity and inhibit inflammation by regulating intestinal microbiome (D’Onofrio et al., 2021; Zhuang et al., 2022). ILA significantly reduced LPS-induced macrophage inflammatory response and oxidative stress response, and is elevated in infant feces, showing protective effect on intestinal epithelial cells (Ehrlich et al., 2020; Meng et al., 2020). In addition, butyrate supplements have been shown to reduce the severity of inflammation in arthritis mice by increasing levels of 5-HIAA (Rosser et al., 2020). In this study, we found the disordered indoles and their derivatives in ALI mice in the metabolomic analysis of cecal contents, and RRTP reversed this disturbance. Characteristically, RRTP significantly increased the intestinal microbiota-derived tryptophan metabolites described above, suggesting that RRTP can promote the remission of lung tissue injury by improving dysregulated metabolism in ALI mice through regulation of intestinal flora.

**Figure 7:**
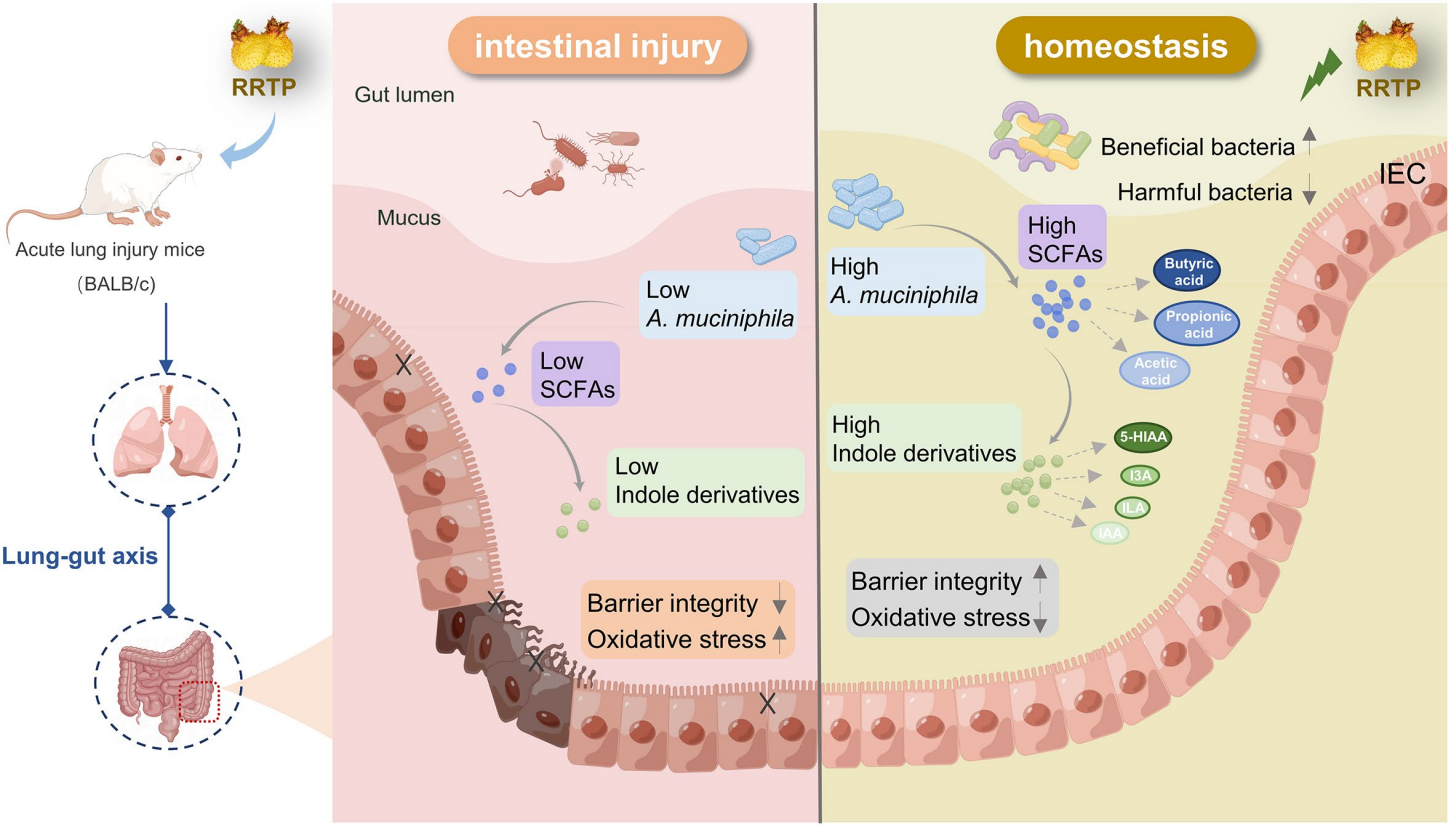
Schematic diagram representing the amelioration mechanism of RRTP improving intestinal homeostasis in LPS-induced ALI mice.

Cumulatively, RRTP is the active components of a promising underutilized functional food source that can synergistically exert anti-ALI efficacy by significantly ameliorating intestinal tissue damage, inhibiting oxidative stress, increasing SCFAs in cecal contents, regulating the composition and structure of intestinal flora, increasing *A. muciniphila* and modulating disordered intestinal endogenous metabolites (Figure 7).

## 4 Conclusion

This study showed that RRTP has significant advantages in adjuvant therapy of ALI, and systematically clarified its comprehensive improvement mechanism from a new perspective of “lung-gut axis,” which provides a breakthrough for the food and healthcare industries to develop products from botanical functional herbs and foods to prevent or alleviate ALI by regulating intestinal flora.

## Data availability statement

The original contributions presented in the study are included in the article/Supplementary material, further inquiries can be directed to the corresponding author.

## Ethics statement

Ethical approval was not required for the studies on animals in accordance with the local legislation and institutional requirements because only commercially available established cell lines were used.

## Author contributions

LT: Data curation, Validation, Writing – original draft, Writing – review & editing. SZ: Conceptualization, Writing – original draft. MZ: Formal analysis, Data curation, Writing – review & editing. PW: Supervision, Writing – review & editing. GL: Software, Writing – review & editing. ZG: Conceptualization, Writing – original draft. XG: Funding acquisition, Project administration, Writing – review & editing.

## References

[ref100] Dang A. T. Marsland B. J.. (2019). Microbes, metabolites, and the gut-lung axis. Mucosal Immunology 12:843–850. doi: 10.1038/s41385-019-0160-6 30976087

[ref1] D’Onofrio F. Renga G. Puccetti M. Pariano M. Bellet M. M. Santarelli I.. (2021). Indole-3-carboxaldehyde restores gut mucosal integrity and protects from liver fibrosis in murine sclerosing cholangitis. Cells 10:1622. doi: 10.3390/cells10071622, PMID: 34209524 PMC8305598

[ref2] Day B. J. (2009). Catalase and glutathione peroxidase mimics. Biochem. Pharmacol. 77, 285–296. doi: 10.1016/j.bcp.2008.09.029, PMID: 18948086 PMC2657365

[ref3] de Vos W. M. Tilg H. Van Hul M. Cani P. D. (2022). Gut microbiome and health: mechanistic insights. Gut 71, 1020–1032. doi: 10.1136/gutjnl-2021-326789, PMID: 35105664 PMC8995832

[ref4] Ehrlich A. M. Pacheco A. R. Henrick B. M. Taft D. Xu G. Huda M. N.. (2020). Indole-3-lactic acid associated with *Bifidobacterium*-dominated microbiota significantly decreases inflammation in intestinal epithelial cells. BMC Microbiol. 20:357. doi: 10.1186/s12866-020-02023-y, PMID: 33225894 PMC7681996

[ref5] Frati F. Salvatori C. Incorvaia C. Bellucci A. Di Cara G. Marcucci F.. (2018). The role of the microbiome in asthma: the gut-lung axis. Int. J. Mol. Sci. 20:123. doi: 10.3390/ijms20010123, PMID: 30598019 PMC6337651

[ref6] Gorman E. A. O’kane C. M. Mcauley D. F. (2022). Acute respiratory distress syndrome in adults: diagnosis, outcomes, long-term sequelae, and management. Lancet 400, 1157–1170. doi: 10.1016/S0140-6736(22)01439-8, PMID: 36070788

[ref7] He Y.-Q. Zhou C.-C. Yu L.-Y. Wang L. Deng J.-L. Tao Y.-L.. (2021). Natural product derived phytochemicals in managing acute lung injury by multiple mechanisms. Pharmacol. Res. 163:105224. doi: 10.1016/j.phrs.2020.105224, PMID: 33007416 PMC7522693

[ref8] Hou H. Chen D. Zhang K. Zhang W. Liu T. Wang S.. (2022). Gut microbiota-derived short-chain fatty acids and colorectal cancer: ready for clinical translation? Cancer Lett. 526, 225–235. doi: 10.1016/j.canlet.2021.11.027, PMID: 34843863

[ref9] Huang C. Du W. Ni Y. Lan G. Shi G. (2022). The effect of short-chain fatty acids on M2 macrophages polarization *in vitro* and *in vivo*. Clin. Exp. Immunol. 207, 53–64. doi: 10.1093/cei/uxab028, PMID: 35020860 PMC8802183

[ref10] Huang X. Fan X. Ying J. Chen S. (2019). Emerging trends and research foci in gastrointestinal microbiome. J. Transl. Med. 17:67. doi: 10.1186/s12967-019-1810-x, PMID: 30819194 PMC6396506

[ref11] Iddrisu I. Soumyakrishnan S. Joseph A. A. Xu J. Boakai R. Sreepriya M.. (2022). Modulatory effect of gut microbiota on the gut-brain, gut-bone axes, and the impact of cannabinoids. Metabolites 12:1247. doi: 10.3390/metabo12121247, PMID: 36557285 PMC9781427

[ref12] Jandhyala S. M. Talukdar R. Subramanyam C. Vuyyuru H. Sasikala M. Nageshwar Reddy D. (2015). Role of the normal gut microbiota. World J. Gastroenterol. 21, 8787–8803. doi: 10.3748/wjg.v21.i29.8787 26269668 PMC4528021

[ref13] Ji J. Zhang S. Yuan M. Zhang M. Tang L. Wang P.. (2022). Fermented *Rosa roxburghii* Tratt juice alleviates high-fat diet-induced hyperlipidemia in rats by modulating gut microbiota and metabolites. Front. Pharmacol. 13:883629. doi: 10.3389/fphar.2022.883629, PMID: 35668952 PMC9164371

[ref14] Kawanishi S. Ohnishi S. Ma N. Hiraku Y. Oikawa S. Murata M. (2017). Nitrative and oxidative DNA damage in infection-related carcinogenesis in relation to cancer stem cells. Genes Environ. 38:26. doi: 10.1186/s41021-016-0055-7 28050219 PMC5203929

[ref15] Kellner M. Noonepalle S. Lu Q. Srivastava A. Zemskov E. Black S. M. (2017). ROS signaling in the pathogenesis of acute lung injury (ALI) and acute respiratory distress syndrome (ARDS). Adv. Exp. Med. Biol. 967, 105–137. doi: 10.1007/978-3-319-63245-2_8 29047084 PMC7120947

[ref16] Markowiak-Kopeć P. Śliżewska K. (2020). The effect of probiotics on the production of short-chain fatty acids by human intestinal microbiome. Nutrients 12:1107. doi: 10.3390/nu12041107, PMID: 32316181 PMC7230973

[ref17] Meng M. M. Jia Y. K. Shuang J. Yuan Y. W. Si L. W. Jing J. Y.. (2022). Melatonin suppresses macrophage M1 polarization and ROS-mediated pyroptosis via activating ApoE/LDLR pathway in influenza A-induced acute lung injury. Oxidative Med. Cell. Longev. 2022:2520348. doi: 10.1155/2022/2520348, PMID: 36425057 PMC9681554

[ref18] Meng D. Sommella E. Salviati E. Campiglia P. Ganguli K. Djebali K.. (2020). Indole-3-lactic acid, a metabolite of tryptophan, secreted by *Bifidobacterium longum* subspecies infantis is anti-inflammatory in the immature intestine. Pediatr. Res. 88, 209–217. doi: 10.1038/s41390-019-0740-x 31945773 PMC7363505

[ref19] Miao L. St. Clair D. K. (2009). Regulation of superoxide dismutase genes: implications in disease. Free Radic. Biol. Med. 47, 344–356. doi: 10.1016/j.freeradbiomed.2009.05.018, PMID: 19477268 PMC2731574

[ref20] Ornatowski W. Lu Q. Yegambaram M. Garcia A. E. Zemskov E. A. Maltepe E.. (2020). Complex interplay between autophagy and oxidative stress in the development of pulmonary disease. Redox Biol. 36:101679. doi: 10.1016/j.redox.2020.101679, PMID: 32818797 PMC7451718

[ref21] Ouyang J. Lin J. Isnard S. Fombuena B. Peng X. Marette A.. (2020). The bacterium *Akkermansia muciniphila*: a sentinel for gut permeability and its relevance to HIV-related inflammation. Front. Immunol. 11:645. doi: 10.3389/fimmu.2020.00645, PMID: 32328074 PMC7160922

[ref22] Rosser E. C. Piper C. J. M. Matei D. E. Blair P. A. Rendeiro A. F. Orford M.. (2020). Microbiota-derived metabolites suppress arthritis by amplifying aryl-hydrocarbon receptor activation in regulatory B cells. Cell Metab. 31, 837–851.e10. doi: 10.1016/j.cmet.2020.03.003, PMID: 32213346 PMC7156916

[ref23] Su X. Gao Y. Yang R. (2022). Gut microbiota-derived tryptophan metabolites maintain gut and systemic homeostasis. Cells 11:2296. doi: 10.3390/cells11152296, PMID: 35892593 PMC9330295

[ref24] Tang J. Xu L. Zeng Y. Gong F. (2021). Effect of gut microbiota on LPS-induced acute lung injury by regulating the TLR4/NF-kB signaling pathway. Int. Immunopharmacol. 91:107272. doi: 10.1016/j.intimp.2020.107272, PMID: 33360370

[ref25] Tang L. Zhang S. Zhang M. Wang P. J. Liang G. Y. Gao X. L. (2022). Analysis of protective effects of *Rosa roxburghii* Tratt fruit polyphenols on lipopolysaccharide-induced acute lung injury through network pharmacology and metabolomics. Food Sci. Nutr. 10, 4258–4269. doi: 10.1002/fsn3.3019, PMID: 36514748 PMC9731534

[ref26] Tang L. Zhang S. Zhang M. Wang P. J. Liang G. Y. Gao X. L. (2023). Integrated proteomics and metabolomics analysis to explore the amelioration mechanisms of *Rosa roxburghii* Tratt fruit polyphenols on lipopolysaccharide-induced acute lung injury mice. J. Agric. Food Chem. 71, 3079–3092. doi: 10.1021/acs.jafc.2c04344, PMID: 36745194

[ref27] Tatsumi Y. Kato A. Niimi N. Yako H. Himeno T. Kondo M.. (2022). Docosahexaenoic acid suppresses oxidative stress-induced autophagy and cell death via the AMPK-dependent signaling pathway in immortalized Fischer rat Schwann cells 1. Int. J. Mol. Sci. 23:4405. doi: 10.3390/ijms23084405, PMID: 35457223 PMC9027959

[ref28] Thalacker-Mercer A. E. Gheller M. E. (2020). Benefits and adverse effects of histidine supplementation. J. Nutr. 150, 2588S–2592S. doi: 10.1093/jn/nxaa229, PMID: 33000165

[ref29] Wang L. Li C. Huang Q. Fu X. (2020). Polysaccharide from *Rosa roxburghii* Tratt fruit attenuates hyperglycemia and hyperlipidemia and regulates colon microbiota in diabetic db/db mice. J. Agric. Food Chem. 68, 147–159. doi: 10.1021/acs.jafc.9b06247 31826616

[ref30] Wang T. Lin S. Liu R. Li H. Liu Z. Zhang X.. (2020). Metabolomic profile perturbations of serum, lung, bronchoalveolar lavage fluid, spleen and feces in LPS-induced acute lung injury rats based on HPLC-ESI-QTOF-MS. Anal. Bioanal. Chem. 412, 1215–1234. doi: 10.1007/s00216-019-02357-1 31940090

[ref31] Wang Z. Liu J. Li F. Luo Y. Ge P. Zhang Y.. (2022). The gut-lung axis in severe acute pancreatitis-associated lung injury: the protection by the gut microbiota through short-chain fatty acids. Pharmacol. Res. 182:106321. doi: 10.1016/j.phrs.2022.106321 35752356

[ref32] Wang L. T. Lv M. J. An J. Y. Fan X. H. Dong M. Z. Zhang S. D.. (2021). Botanical characteristics, phytochemistry and related biological activities of *Rosa roxburghii* Tratt fruit, and its potential use in functional foods: a review. Food Funct. 12, 1432–1451. doi: 10.1039/d0fo02603d, PMID: 33533385

[ref33] Wang M. Ma L.-J. Yang Y. Xiao Z. Wan J.-B. (2019). n-3 Polyunsaturated fatty acids for the management of alcoholic liver disease: a critical review. Crit. Rev. Food Sci. Nutr. 59, S116–S129. doi: 10.1080/10408398.2018.1544542, PMID: 30580553

[ref34] Wedgwood S. Gerard K. Halloran K. Hanhauser A. Monacelli S. Warford C.. (2020). Intestinal dysbiosis and the developing lung: the role of toll-like receptor 4 in the gut-lung axis. Front. Immunol. 11:357. doi: 10.3389/fimmu.2020.00357, PMID: 32194566 PMC7066082

[ref35] Wu Z. Huang S. Li T. Li N. Han D. Zhang B.. (2021). Gut microbiota from green tea polyphenol-dosed mice improves intestinal epithelial homeostasis and ameliorates experimental colitis. Microbiome 9:184. doi: 10.1186/s40168-021-01115-9, PMID: 34493333 PMC8424887

[ref36] Yoo J. Y. Groer M. Dutra S. V. Sarkar A. McSkimming D. I. (2020). Gut microbiota and immune system interactions. Microorganisms 8:1587. doi: 10.3390/microorganisms8101587 33076307 PMC7602490

[ref37] Younes N. B. Mohamed O. A. Rizk N. M. (2022). Docosahexaenoic acid counteracts the hypoxic-induced inflammatory and metabolic alterations in 3T3-L1 adipocytes. Nutrients 14:4600. doi: 10.3390/nu14214600, PMID: 36364860 PMC9659308

[ref38] Zafar H. Saier M. H. Jr. (2021). Gut *Bacteroides* species in health and disease. Gut Microbes 13, 1–20. doi: 10.1080/19490976.2020.1848158, PMID: 33535896 PMC7872030

[ref39] Zhang T. Li Q. Cheng L. Buch H. Zhang F. (2019). *Akkermansia muciniphila* is a promising probiotic. Microb. Biotechnol. 12, 1109–1125. doi: 10.1111/1751-7915.13410 31006995 PMC6801136

[ref40] Zhuang H. Li B. Xie T. Xu C. Ren X. Jiang F.. (2022). Indole-3-aldehyde alleviates chondrocytes inflammation through the AhR-NF-κB signalling pathway. Int. Immunopharmacol. 113:109314. doi: 10.1016/j.intimp.2022.109314, PMID: 36252481

