# Supplementary material for: Unlocking the potential of Rosa roxburghii Tratt polyphenol: a novel approach to treating acute lung injury from a perspective of the lung-gut axis
Source: Front Microbiol. 2024 Jan 11;15:1351295. doi: 10.3389/fmicb.2024.1351295 (PMC10809152; doi:10.3389/fmicb.2024.1351295)

**Supplementary material**

**Unlocking the potential of *Rosa Roxburghii* Tratt polyphenol: a novel approach to treating acute lung injury from a perspective of the lung-gut axis**

**Li Tang, Shuo Zhang, Min Zhang, Peng-Jiao Wang, Gui-You Liang, Zhitong Gan and Xiu-Li Gao^*^**

*** Correspondence:**

**Corresponding author**

Xiu-li Gao, Professor

State Key Laboratory of Functions and Applications of Medicinal Plants & School of Pharmacy, Guizhou Medical University, Guiyang 550025, PR China.

Tel. / fax: (+86) 0851 88416154

E-mail: gaoxl@gmc.edu.cn

**Supplementary Figure 1.** OPLS-DA score plots between the model group and the RRTP group of the cecal contents metabolomics analysis.


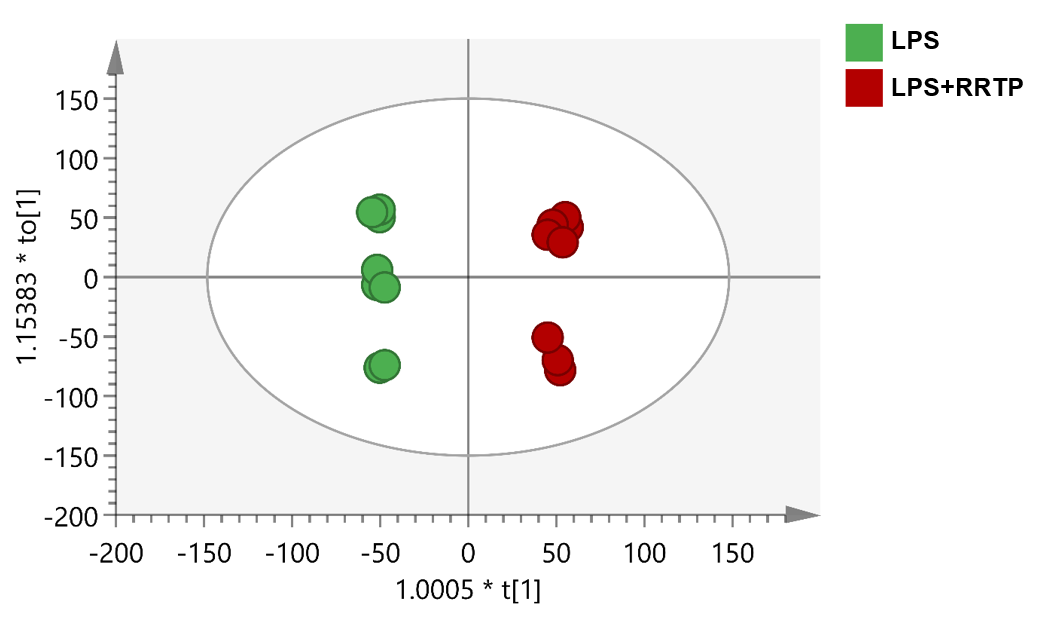


**Supplementary Figure 2.** OPLS-DA loading plots between the model group and the RRTP group of the cecal contents metabolomics analysis.


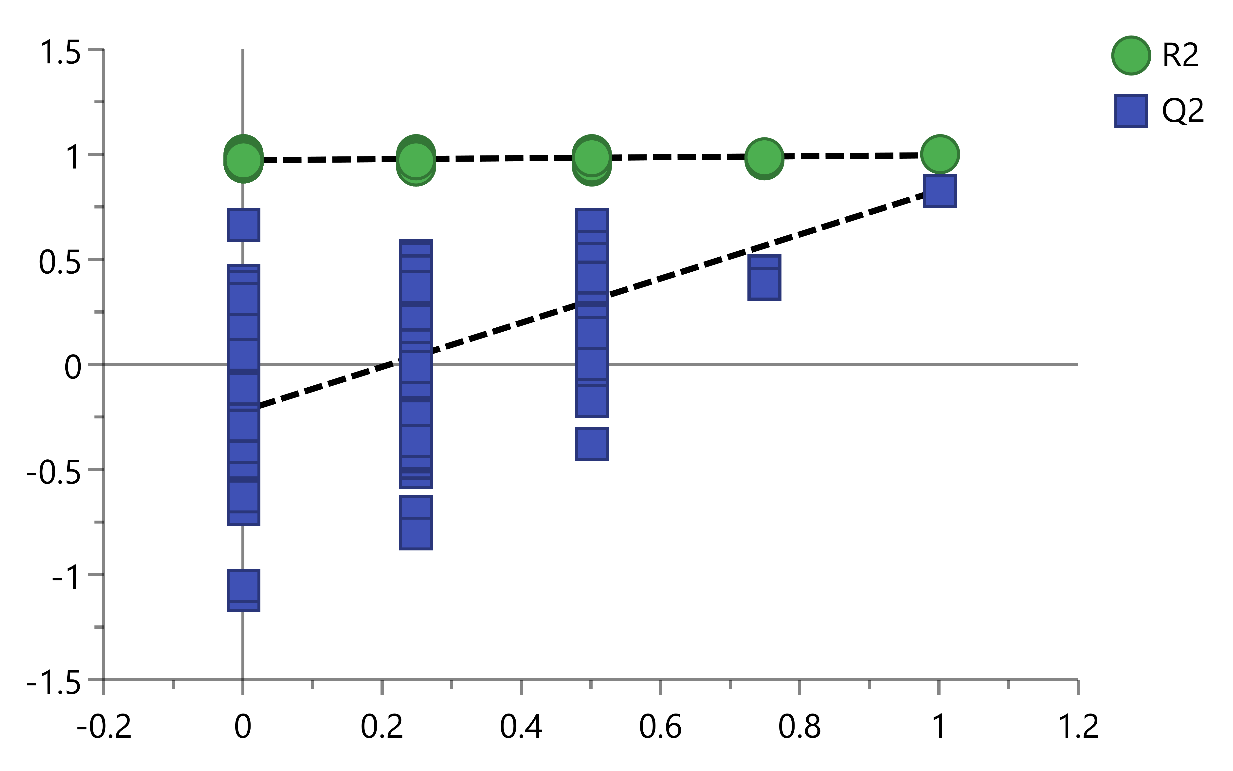

Supplement: Supplementary file 1 [file Data_Sheet_1.docx]
